# Child Injury Prevention: An Overlooked Challenge for Child Survival

**DOI:** 10.3390/ijerph10020568

**Published:** 2013-02-01

**Authors:** David Meddings

**Affiliations:** Department of Violence and Injury Prevention and Disability, Noncommunicable Diseases and Mental Health, World Health Organization, 20 Avenue Appia, CH-1211 Geneva 27, Switzerland

Those interested in child survival frequently cite and follow the under-five mortality rate. While a useful measure, the under-five mortality rate lumps together neonatal and post-neonatal mortality with deaths in the 1–4 year period. Unfortunately, this hampers public health decision making about the most appropriate child survival strategies as children survive beyond their first year of life.

The first year of life is unquestionably a risky one in many parts of the world, with the neonatal period being particularly fraught with danger. An assessment of under-five deaths in 187 countries published in 2010 estimated over 40% of the 7.7 million under-five deaths in 2010 occurred during the first 27 days, with another 30% occurring from the 28th to 364th day of life [1]. Analyses show the main drivers of neonatal deaths are sepsis, birth asphyxia, congenital abnormalities and preterm complications, whereas deaths occurring in the post-neonatal period most frequently arise from infectious conditions such as pneumonia and diarrhoea [2].

This special issue on child injury prevention highlights a range of issues and injury mechanisms which progressively account for a greater proportion of child mortality following infancy. This attention is warranted, as the global patterns of child survival have undergone important, but largely unrecognized changes over the last decades.

An analysis of 1–4 year mortality in Bangladesh within a cohort of 8,000 rural children and a nationwide survey sample identified drowning as a major mortality threat, accounting for 43% of deaths among the rural child cohort and 20% in the survey population [3]. An assessment of under-five mortality in China found injury causes to be the leading cause of death among 1–4 year olds, accounting for 38% of deaths [4].

These are illustrative examples that should be seen within the context of a larger shift in global mortality patterns. In 2011, an examination of mortality trends among 1–24 year olds over 50 years which drew on data from 50 countries found mortality improvements amongst 1–4 year olds of between 85–93% over the 50 years ending in 2004, and that these declines were largely due to improvements in mortality related to communicable diseases [5]. The authors noted that, while mortality for injury related causes also improved over this period, the improvements were of smaller magnitude, ranging from 55–75%. Indeed, the differences in mortality declines were sufficiently great that the study revealed a reversal in historical mortality patterns, with mortality amongst 1–4 year olds falling below that for 20–24 year old males, because of static or rising injury-related mortality, particularly in young men.

The relevance of this for the child survival agenda and global public health more broadly should be clear. While the first year of life remains an inherently risky one that must remain a public health priority, the substantial majority of children who survive this first year are confronted by a wider range of health threats than perinatal complications and communicable disease. Furthermore, the injury-related mechanisms among these continue to grow in importance as children pass into adolescence and adulthood. There are vastly different public health programming and policy response implications for addressing violence prevention, road safety, or drowning than for dealing with vaccine-preventable disease, pneumonia or reproductive health.

Governments seeking to provide for the curative and preventive health needs of their populations need to cater to these throughout the life cycle. As children move from infancy to older ages their threats to health do not just recede; they undergo a qualitative shift which includes an increasing preponderance of injury threats. The current palette of child survival programming has not evolved in any substantive way to address these.

In 2008, the joint WHO/UNICEF *World Report on Child Injury Prevention* provided an overview of child injury worldwide, and set out the evidence on the effectiveness of different interventions to prevent child injury. A notable feature of interventions to prevent child injury is that many of them are highly cost effective. Indeed, the report showed clearly that implementation of strategies such as vehicle child-restraints, isolation fencing of swimming pools, laws on smoke alarms and hot tap water temperature, window guards and child-resistant packaging of medicines and poisons had been associated with dramatic and sustained reductions in child injury in many countries. Further research, which the report also called for, will do much to add to the evidence base for effective child injury prevention.

The report also put forward recommendations for international, development and donor organizations to improve child injury prevention. In 2011 the sixty-fourth World Health Assembly adopted a resolution on child injury prevention which welcomed the above report and its recommendations. Among other things, the World Health Assembly resolution states failure to address child injury will hinder achievement of Millennium Development Goal 4, urges Member States to include child injury prevention within child survival programmes, and to implement as appropriate the recommendations of the *World Report on Child Injury Prevention,* including assigning a leadership role within government for child injury prevention. The resolution also calls on the World Health Organization to collaborate with organizations of the United Nations system, international development partners and nongovernmental organizations to establish a network for the effective coordination and implementation of activities for child injury prevention in low- and middle-income countries. WHO is in the process of establishing this network now, and hopes that the network will increase visibility of child injury, as well as improving the coordination and communication amongst actors engaged in child injury prevention.

This special issue is a timely and important reminder of the myriad ways in which child injury has emerged as a public health concern for child survival that can, and should, no longer be ignored. Injuries to children are largely preventable, and much greater effort needs to be made to the application of evidence-based child injury prevention in low- and middle-income countries. Those in public health who wish to see progress on child survival continue must recognize the changing nature of child mortality patterns and adapt to this. Integrating child injury prevention within child survival programming can emphasize the importance of the child survival agenda as a whole and make it more reflective of the full breadth of threats to children’s health.
